# Integrating Taguchi Method and Gray Relational Analysis for Auto Locks by Using Multiobjective Design in Computer-Aided Engineering

**DOI:** 10.3390/polym14030644

**Published:** 2022-02-08

**Authors:** Wei-Tai Huang, Zi-Yun Tasi, Wen-Hsien Ho, Jyh-Horng Chou

**Affiliations:** 1 Department of Mechanical Engineering, National Pingtung University of Science and Technology, Pingtung 912, Taiwan; weitai@g4e.npust.edu.tw (W.-T.H.); zyt.st11@gmail.com (Z.-Y.T.); 2 Department of Medical Research, Kaohsiung Medical University Hospital, Kaohsiung 807, Taiwan; 3 Department of Healthcare Administration and Medical Informatics, Kaohsiung Medical University, Kaohsiung 807, Taiwan; 4 Department of Mechanical and Computer-Aided Engineering, Feng Chia University, Taichung 407, Taiwan

**Keywords:** injection molding, Taguchi method, gray relational analysis, warpage, temperature distribution, conformal cooling

## Abstract

In automobiles, lock parts are matched with inserts, and this is a crucial quality standard for the dimensional accuracy of the molding. This study employed moldflow analysis to explore the influence of various injection molding process parameters on the warpage deformation. Deformation of the plastic part is caused by the nonuniform product temperature distribution in the manufacturing process. Furthermore, improper parameter design leads to substantial warpage and deformation. The Taguchi robust design method and gray correlation analysis were used to optimize the process parameters. Multiobjective quality analysis was performed for achieving a uniform temperature distribution and reducing the warpage deformation to obtain the optimal injection molding process parameters. Subsequently, three water cooling system designs—original cooling, U-shaped cooling, and conformal cooling—were tested to modify the temperature distribution and reduce the warpage. Taguchi gray correlation analysis revealed that the main influencing parameter was the mold temperature followed by the holding pressure. Moreover, the results indicated that the conformal cooling system improved the average temperature distribution.

## 1. Introduction

With developments in the plastics industry, injection molding has become the most widely used technique for molding plastic, with most plastic products manufactured using this approach. This technique affords excellent dimensional accuracy, stability, and surface accuracy. The main factor influencing the quality of injection-molded products is the selection of the process parameters. The product quality varies with different process settings and conditions. Therefore, the selection and setting of suitable process parameters is crucial in injection molding. The process parameters for mold production are typically established through trial-and-error or heuristic rules. This approach hampers quality improvement. Therefore, in this study, we adopted a systematic modeling approach to perform the single- and multiobjective quality optimization of the process parameters used in injection molding.

Technological developments have resulted in the introduction of computer-aided design and computer-aided engineering (CAE) simulation methods to assist developers in analyzing and predicting problems relating to injection molding and production. These technologies help to reduce the number of trials required and to maximize product quality. In this study, we performed a moldflow analysis (Moldex3D) using CAE. In CAE, the finite element method (FEM) is generally used to simulate the various material conditions in the mold cavity during plastic injection molding. The simulation results can serve as a reference for establishing the injection molding parameters and model design, thereby stimulating product development and reducing production and mold design costs [1]. Currently, plastic injection molding is widely used in the manufacturing of automotive parts and components such as bumpers, lights, dashboards, and connectors. In particular, auto lock parts are structurally complex and require high fitting accuracy. Finished lock parts often exhibit warpage deformation, volume shrinkage, and suture flaws [2]. Therefore, the selection and setting of the process parameters are vital.

Rosaa et al. [3] advocated the wide use of experimental design for optimizing the molding parameters and thereby improving product quality. The Taguchi method can be effectively used to reduce the number of tests required, thereby enhancing the test efficiency. Gu et al. [4] applied the Taguchi robust design method to analyze the injection molding process of recycled plastic (specifically, polypropylene). Their findings validated that optimizing the process parameters effectively improved the mechanical performance. Wang et al. [5] applied the Taguchi robust design method to examine the effects of plastic valves on the optimization of the process parameter design. The results of an FEM CAE analysis indicated that the mold temperature was the primary factor influencing molding. Marinset al. [6] applied the Taguchi method and conducted an analysis of variance (ANOVA) to examine the flaws of injection molding and to evaluate the effects of various injection molding parameters on warpage and shrinkage. They found that the holding time and holding pressure were the key factors influencing warpage and bending. Chen and Huang [7] integrated the analytic hierarchy process and Taguchi method to investigate injection molding warpage. They used the Taguchi design data to analyze four factors—injection pressure, holding pressure, holding time, and mold temperature—and they determined the optimal parameter combination to minimize warpage.

Chang et al. [8] adopted gray relational analysis combined with a fuzzy method to optimize the process parameters for manufacturing cellphone cases. The results of a finite element analysis indicated that the mold temperature and holding pressure were the main factors influencing the volume shrinkage and temperature distribution. Lin and Chen [9] applied the Taguchi method and gray relational analysis to analyze the multiobjective optimization of injection-molded plastic lenses. Their simulation results confirmed that the joint optimization process yielded an effective improvement in the quality of the injection-molded lens. Sreedharan et al. [10] used gray relational analysis to achieve multiobjective optimization for multistage sequential plastic injection molding. Their experimental results indicated that the optimal settings produced the expected responses.

Ahn [11] examined different processes for producing conformal cooling channel molds and analyzed the thermal transfer of various conformal cooling channels. Juan et al. [12] compared the cooling channels of thin-walled products produced automatically and manually using software. Cooling channels that were manually designed based on the product shape exhibited significantly less warpage. Wang et al. [13] examined the incorporation of a cooling channel design within a complex automotive part. Subsequently, they analyzed and tested the modified cooling channel design. They found that a uniform mold temperature distribution was achieved and that the surface accuracy of the plastic part was enhanced.

In this study, we examined an auto lock part production line. Warpage deformation was a major problem in the production process, causing misalignment and inaccurate assembly. We combined CAE software with a smart modeling process to address this problem. First, we conducted a CAE moldflow analysis and adopted the Taguchi robust design method to identify a suitable parameter combination to optimize individual single-quality factors and to examine the warpage deformation volume and average temperature. Subsequently, we examined the Taguchi experiment data and conducted a gray relational analysis to identify the optimal parameter combination for the multiobjective quality process. Next, we compared the warpage deformation volume and the average temperature of the modified process with those of the original process. Finally, we incorporated the optimal parameter combinations for the multiobjective quality process into several cooling channel system designs—original cooling, square cooling and conformal cooling—for comparison and analysis.

## 2. Experimental

### 2.1. Construction of Auto Lock Spare Parts

**Figure 1:**
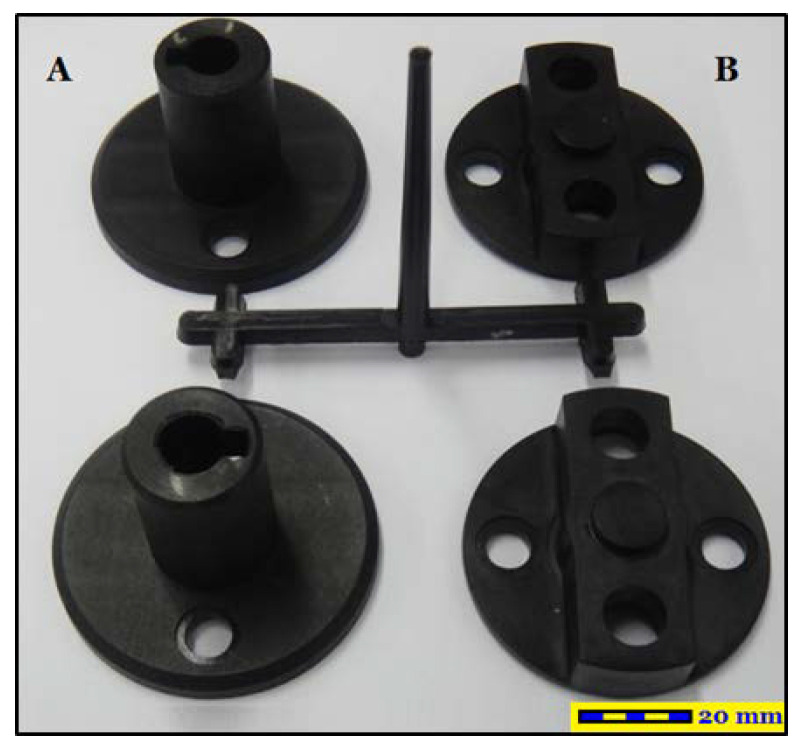
Photograph of a Car Lock Part. (**A**) Part A with the diameter and height 58 mm and 39.20 mm and (**B**) Part B with the diameter and height 54.95 and 18.11 mm.

**Figure 2:**
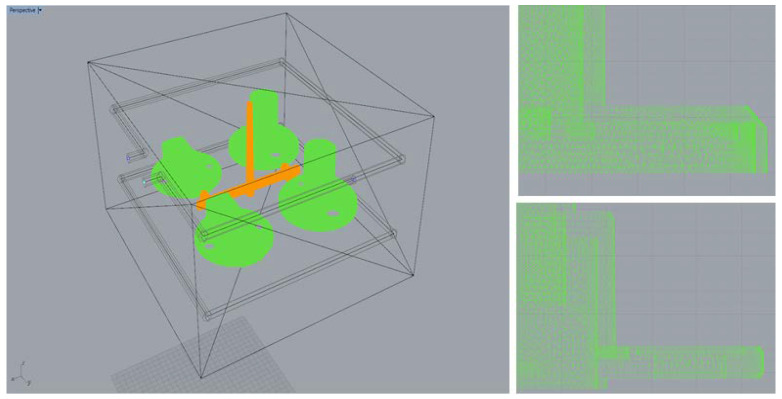
Mesh Figure of a Car Lock Part.

Figure 1 displays the shape of the auto lock parts examined in this study. The original design had a four-cavity configuration. The diameter and height of Part A were 58 mm and 39.20 mm, respectively, and those of Part B were 54.95 and 18.11 mm, respectively. The mold material was NAK80. The injection molding process was simulated using polyamide (PA66). This material exhibits excellent tensile strength, impact resistance, self-lubrication, and abrasion resistance. Owing to its excellent mechanical and thermal resistance, favorable barrier properties, and recyclability [14], PA66 is widely used in automotive parts and components. Table 1 lists the basic characteristics of PA66. Moldex3D was adopted as the CAE software in this study, and the Moldex3D/Solid module and Moldex3D-Mesh module were adopted as the primary and secondary analysis tools, respectively. The mesh comprised 750,000 cells and approximately 700,000 nodes (Figure 2). The original cooling (square) system was used in the two experimental stages of Taguchi robust design process and gray relational analysis.

### 2.2. Simulation and Analysis of Original Process Parameters and Comparison of Plastic Products

**Figure 3:**
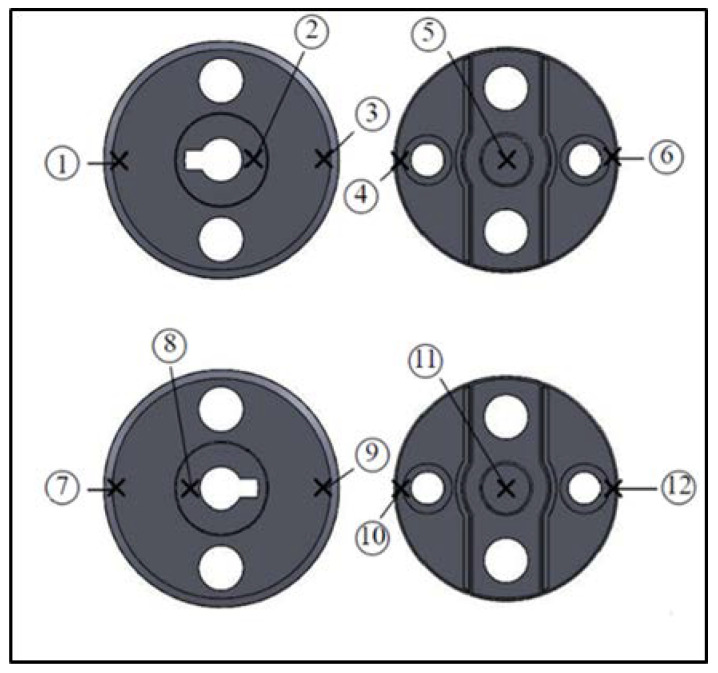
Schematic of Locations of Measurement Points for Car Lock Part.

**Figure 4:**
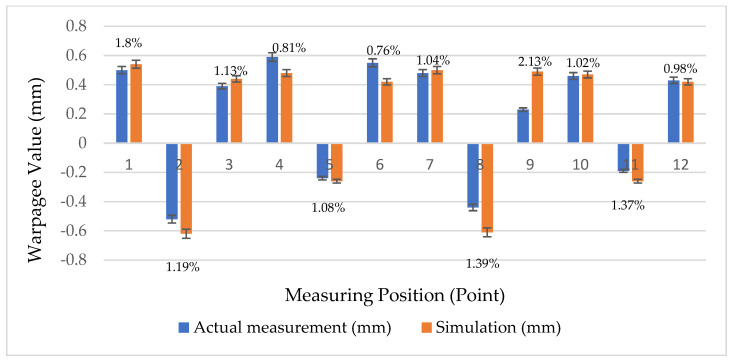
Comparison of Actual and Simulated Measurement Points.

To clarify the status of auto lock parts, we adopted the process parameters provided by the manufacturer as the original ones (Table 2). These were then imported into the CAE software for simulation and analysis. To ensure that the simulations conformed to real-world conditions, we measured the warpage of the product using the Tesa Micro-Hite 3D 4.5.4 coordinate measuring machine with a measurement accuracy of 0.001 mm. Subsequently, we cross-validated the CAE simulation results. Twelve points on the lock part were measured (Figure 3). We then compared the *Z*-axis warpage value of the actual measurements and that of the simulation results (Table 3). The results indicated that the simulation results were highly similar to the actual measurements. The total average comparison error was within 1.16%. The trend chart illustrated in Figure 4 validated that the simulation results were consistent with actual production conditions.

### 2.3. Taguchi Robust Design Process and Gray Relational Analysis

**Figure 5:**
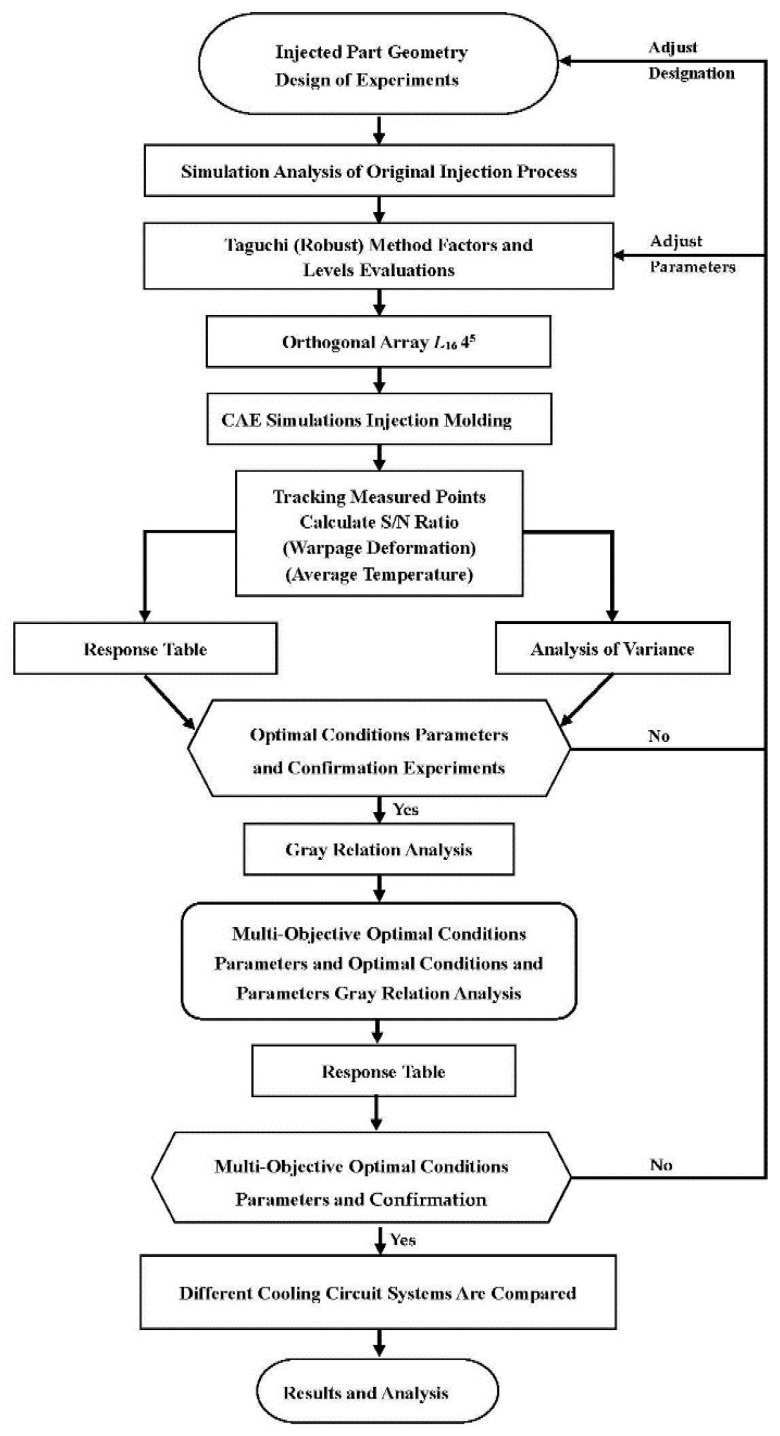
Overall Flowchart of Experiment.

We divided the experimental framework into three parts. In the first part, the Taguchi robust design method was used to examine the warpage and average temperature of the auto lock part and to derive a single-objective optimization design. In the second part, gray relational analysis was conducted. In the third part, the performance of the multicharacteristic optimal parameter combinations in various cooling systems and the effects of these combinations on the warpage and average temperature were compared. Figure 5 presents the overall experimental procedure.

### 2.4. Taguchi Robust Design Process

We selected an *L*_16_(4^5^) orthogonal array for testing. We performed moldflow simulations (Moldex3D) based on the parameter combinations on the orthogonal array and determined the optimal parameters for the injection molding of the auto lock parts on the basis of the signal-to noise (S/N) ratios. The S/N ratios were also used as the ANOVA data to validate the experiment and to determine the factor contribution. Table 4 lists the parameters and levels for the Taguchi robust design method. The warpage and average temperature of the auto lock parts were adopted as the optimal single-objective parameters. The total warpage and average temperature of the auto lock parts were adopted as the optimal performance characteristics. A low performance characteristic value is preferred. Therefore, the quality setting was defined as a static smaller-the-better characteristic.

Taguchi methods are the most widely applied robust design methods in the planning of process parameters [15,16]. The optimization of the injection molded parameters was considered a static problem with smaller-the-better S/N ratios, and it is expressed as:

(1) $$S/N=-10\log_{10}\left[\frac{1}{n}\overset{n}{\underset{i=0}{\sum }}y_{i}^{2}\right]$$

where *n* is the number of instances observed in each experimental combination, and *y_i_* is the ith datum in the experimental combination.

### 2.5. Taguchi Gray Relational Analysis Method

In real-world manufacturing, single-objective quality characteristics cannot satisfy process demands; only multiobjective quality characteristics can. Therefore, the optimization analysis of multiobjective quality parameters was required to achieve the objectives of this study. The Taguchi gray relational analysis method is a multiobjective optimization analysis method. It can accurately improve the quality of multiobjective characteristics [17,18,19]. The S/N ratios obtained using the Taguchi method must be normalized. Therefore, we selected a suitable gray relation molding equation to determine the gray relation coefficients. We also calculated the average values to determine the degree of gray relation among the coefficients. The S/N ratio of each single-quality characteristic was normalized using gray correlation generation, as expressed in Equation (2). The normalized values were between 0 and 1. The normalized data were then incorporated into a gray relational analysis to calculate the gray relational coefficient, as expressed in Equation (3). The mean value of a gray relational coefficient represents a gray relation. Gray relations were calculated using Equation (4) and sorted in descending order.

(2) $$x_{i}^{*}\left(k\right)=\frac{x_{i}^{\left(0\right)}\left(k\right)-min_{all\;i}\left[x_{i}^{\left(0\right)}\left(k\right)\right]}{max_{all\;i}\left[x_{i}^{\left(0\right)}\left(k\right)\right]-min_{all\;i}\left[x_{i}^{\left(0\right)}\left(k\right)\right]},$$

where $x_{i}^{*}\left(k\right)$ represents the gray relational values, and $max_{all\;i}\left[x_{i}^{\left(0\right)}\left(k\right)\right]$ and $min_{all\;i}\left[x_{i}^{\left(0\right)}\left(k\right)\right]$ respectively represent the largest and smallest values in the $x_{i}^{*}\left(k\right)$ sequence.

(3) $$\gamma \left(x_{i}\left(k\right),x_{j}\left(k\right)\right)=\frac{\Delta_{min}+\zeta \Delta_{max}}{\Delta_{0i}\left(k\right)+\zeta \Delta_{max}},$$

where $\gamma \left(x_{i}\left(k\right),x_{j}\left(k\right)\right)$ represents the gray relational coefficients, $\Delta_{0i}\left(k\right)$ represents the sequence differences between corresponding positions in sequence $x_{0}\left(k\right)$ and subsequence $x_{i}\left(k\right)$, and ζ represents the identification coefficient (generally, 0.5).

(4) $$R\left(x_{i},x_{j}\right)=\frac{1}{n}\sum_{k=1}^{n}r(x_{i}\left(k\right),x_{j}\left(k\right)).$$

### 2.6. Comparison and Analysis of Different Cooling Channel Systems

**Figure 6:**
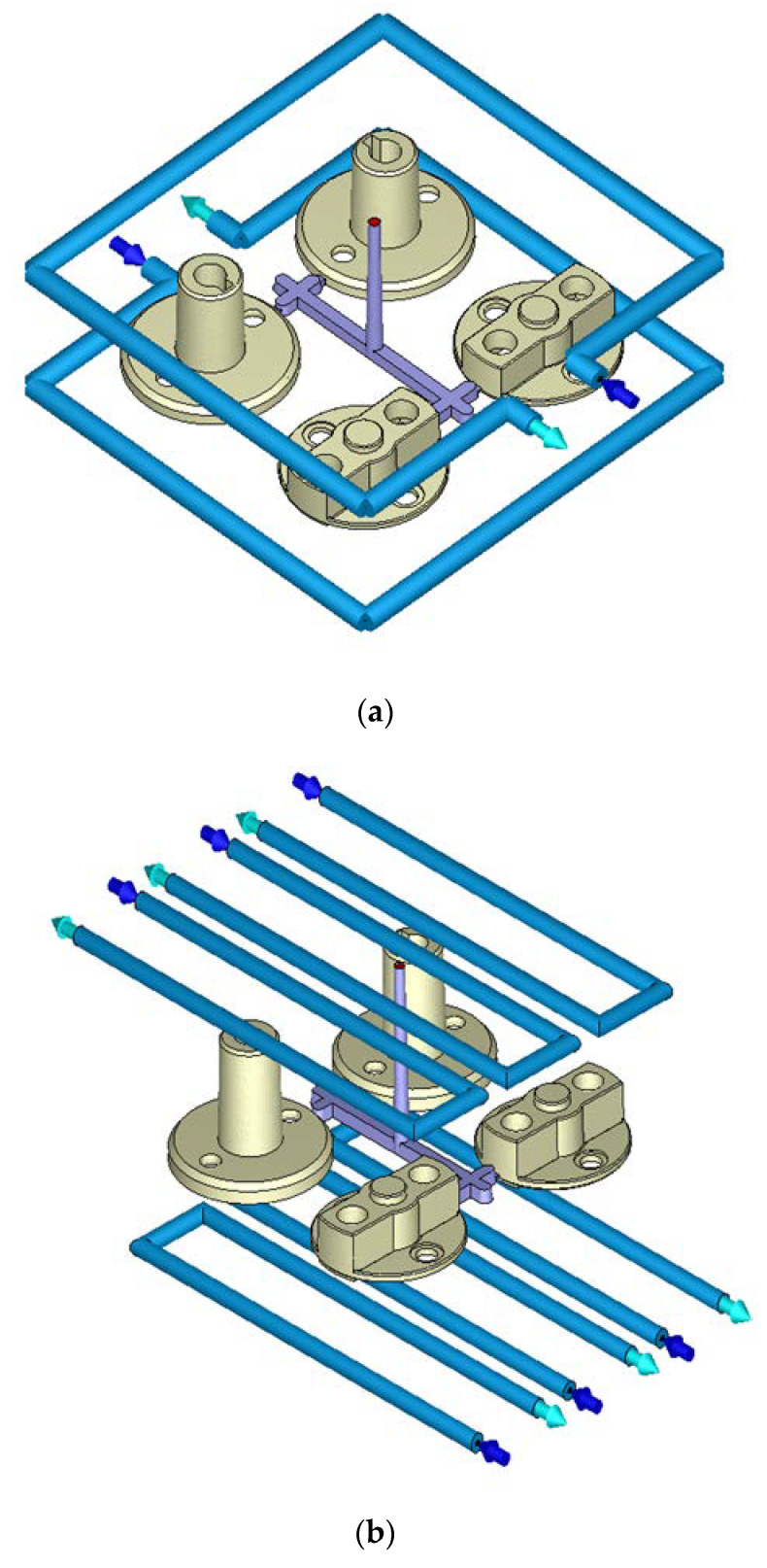

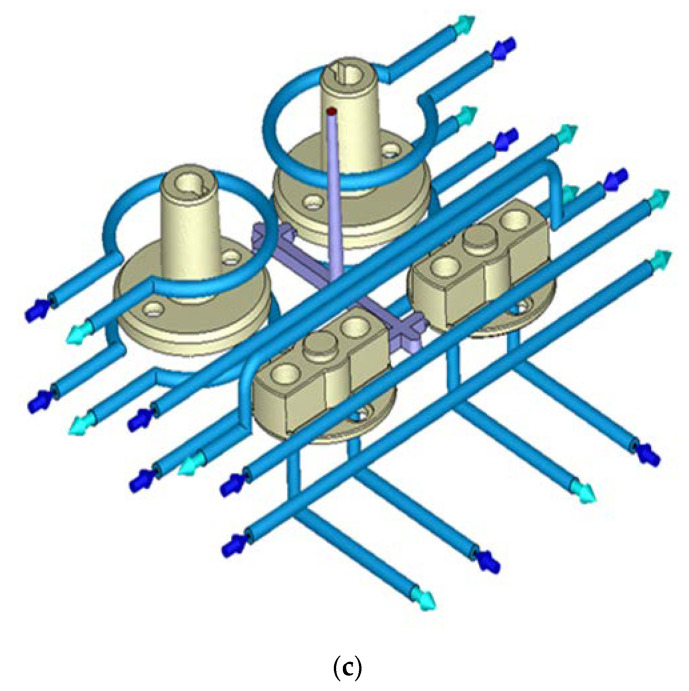
Cooling Configurations. (**a**) Original cooling. (**b**) U-shaped cooling. (**c**) Conformal cooling.

A nonuniform mold temperature distribution causes thermal stress, leading to warpage deformation. Ineffective cooling channel designs not only increase the molding time but also cause uneven cooling, resulting in plastic warpage deformation. In this study, we examined three cooling configurations: original cooling, U-shaped cooling, and conformal cooling, as illustrated in Figure 6. To effectively remove heat, the U-shaped cooling channel design features three cooling channels that are placed above and below the product. In the conformal cooling channel design, cooling channels are placed according to the shape of the auto lock parts, which effectively increases the cooling efficiency [20,21,22,23,24]. The channels surrounded the outer boundaries of the lock parts and were concentrated in regions with slow heat dissipation to enhance cooling efficiency. Table 5 presents a basic comparison of the three cooling channel systems. We observed whether conformal cooling improved the auto lock parts and the effects of conformal cooling on the temperature distribution and warpage of the parts.

## 3. Experimental Results

The experimental results are presented in four parts: optimization of process parameters for warpage deformation volume, optimization of process parameters for temperature distribution, optimal multiobjective quality parameter combination, and comparison and analysis of different cooling channel system designs.

### 3.1. Optimal Process Parameters for Total Warpage Deformation Volume

**Figure 7:**
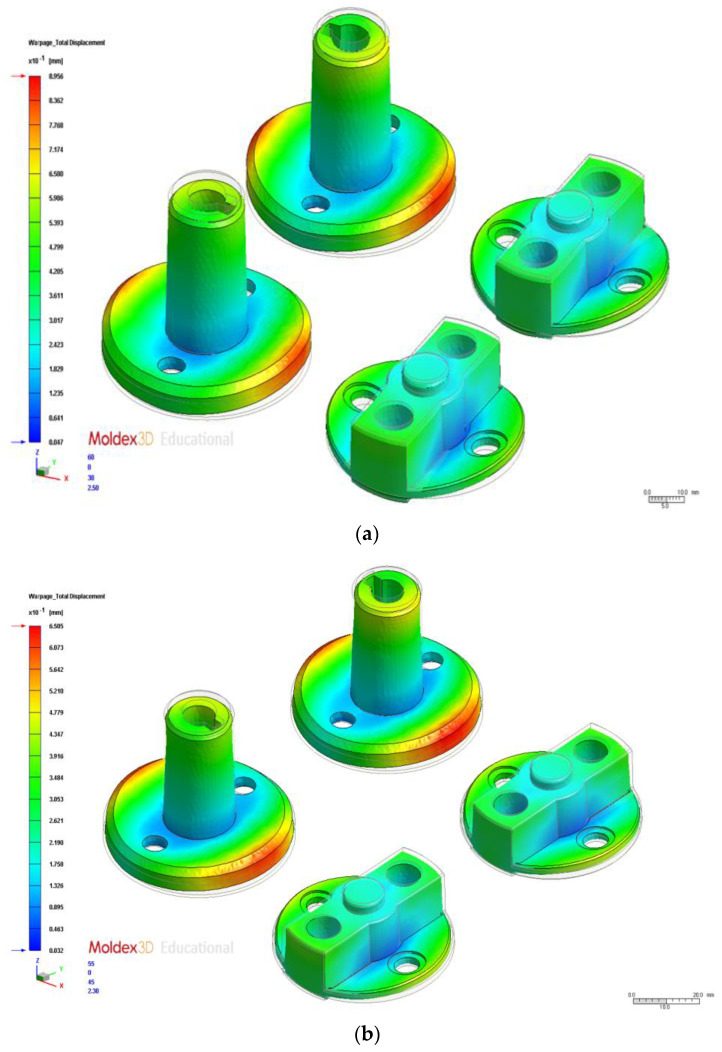
Total Warpage. (**a**) Original process. (**b**) Optimization.

We tested the 16 process parameter combinations in the orthogonal array and examined the warpage deformation volume results to obtain the S/N ratios (Table 6). A total warpage response table for the process parameters at different levels is presented in Table 7. The test results indicated that the optimal process parameter combination was A1B2C2D1E4, where A1 is injection time (0.6 s), B2 is material temperature (255 °C), C2 is mold temperature (75 °C), D1 is injection pressure (120 MPa), and E4 is holding pressure (145 MPa). The optimal total warpage value was 0.61 mm, representing a 0.29-mm increase compared with the original process (Table 8). The total warpage deformation results simulated using the original and optimal process parameters are indicated in Figure 7a,b, respectively. Factor contribution was determined on the basis of the ANOVA results in Table 9. In descending order of contribution, the factors were holding pressure (51.96%), material temperature (22.68%), injection time (14.92%), and mold temperature (7.31%).

### 3.2. Optimal Process Parameters for Average Temperature Difference

**Figure 8:**
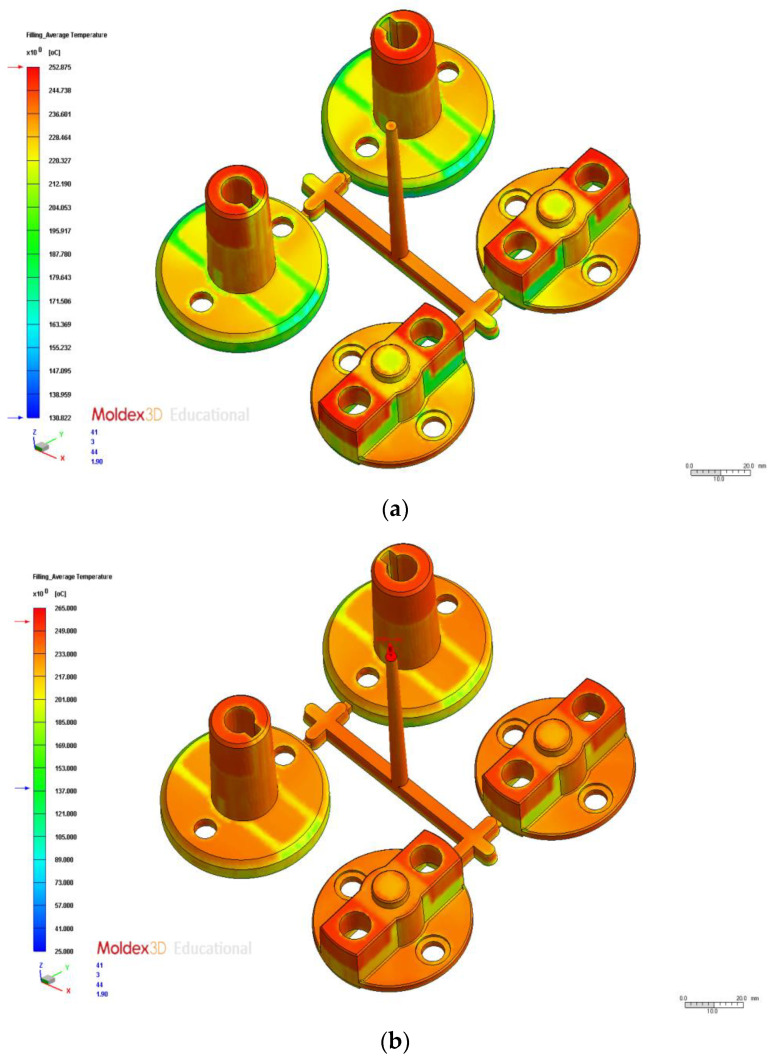
Average Temperature Distribution. (**a**) Original process. (**b**) Optimization.

We tested the 16 process parameter combinations in the orthogonal array and examined the average temperature difference results to obtain the S/N ratios (Table 10). Table 11 presents the average temperature difference response for the process parameters at different levels. The test results indicated that the optimal process parameter combination was A1B1C1D2E1, where A1 is injection time (0.6 s), B1 is material temperature (250 °C), C1 is mold temperature (65 °C), D2 is injection pressure (125 MPa), and E1 is holding pressure (130 MPa). Therefore, this parameter combination was the optimal parameter for the average temperature difference in injection molding, and it reduced the average temperature difference by 6.84 °C compared with the original process parameters (Table 12). The postfill average temperature difference results simulated using the original and optimal process parameters are displayed in Figure 8a,b, respectively. The factor contribution was determined according to the ANOVA results in Table 13. In descending order of contribution, the factors were mold temperature (64.70%), injection pressure (14.75%), material temperature (8.34%), and injection time (6.23%).

### 3.3. Multiobjective Optimization Parameters

To identify the optimal parameters for multiobjective quality characteristics that meet industrial requirements, we combined the gray relational analysis method with the Taguchi robust design method. First, the S/N ratios for the 16 parameter combinations for warpage and average temperature difference in the Taguchi orthogonal array were incorporated into Equation (2) to calculate their gray relations and normalize the data. The S/N ratios were converted into a value between 0 and 1 (Table 14). The normalized S/N ratios for the quality characteristics were then incorporated into Equation (3) to calculate the gray relational coefficients at an identification coefficient of 0.5. Finally, the coefficients were incorporated into Equation (4) to determine the gray relation degrees. The degrees were ordered in descending order (Table 15).

**Figure 9:**
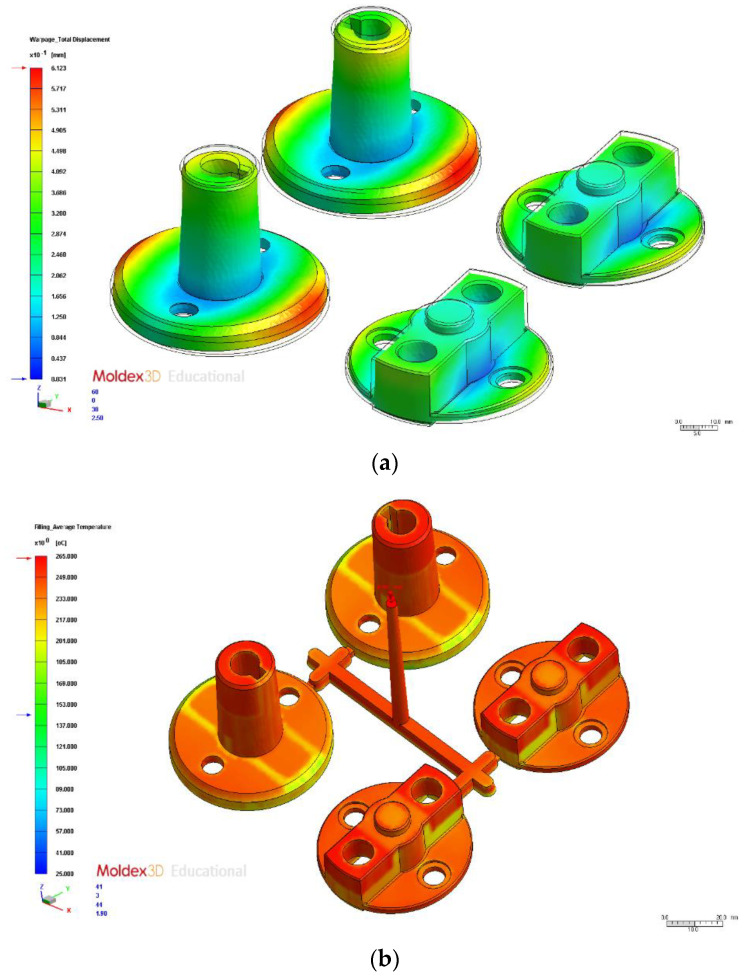
Warpage and Average Temperature Difference with Multiobjective Optimization. (**a**) Warpage with multiobjective optimization. (**b**) Average temperature difference with multiobjective optimization.

The gray relation degrees were consolidated into a response table using various factor levels (Table 16), indicating the changes in the different factors at specific levels. The factor response table and diagram reveal that the optimal process parameter combination for analyzing the multiobjective quality characteristics in the injection molding of auto lock parts was A1B2C1D1E4, where A1 is injection time (0.6 s), B2 is material temperature (260 °C), C1 is mold temperature (65 °C), D1 is injection pressure (120 MPa), and E4 is holding pressure (140 MPa). Because this combination differed from all the other ones in the orthogonal array, it had to be validated and compared with the original process parameter combinations and the single-objective (warpage and average temperature difference) parameter combinations (Table 17). The multiobjective optimization warpage value was 0.62 mm. The warpage results simulated using the original process parameter are presented in Figure 7a, and those simulated using the multiobjective optimization are shown in Figure 9a. The average temperature difference obtained using the multiobjective optimization was 10.16 °C. Compared with the single-objective optimization (warpage 0.61 mm and average temperature difference 7.59 °C, the multiobjective optimization must consider the two-objective optimization characteristics and thus lose some quality characteristics. We compared the results with the two single-objective optimal quality characteristics. We noted a 1.6% loss in warpage quality and a 5.2% loss in average temperature difference quality. The average temperature difference obtained by optimization using the original process parameters is shown in Figure 8a, and that obtained by the multiobjective optimization is shown in Figure 9b. The test results indicated that the parameters obtained using the multiobjective optimization substantially improved the warpage and average temperature difference. However, the multiobjective optimal parameters covered multiple quality characteristics.

**Figure 10:**
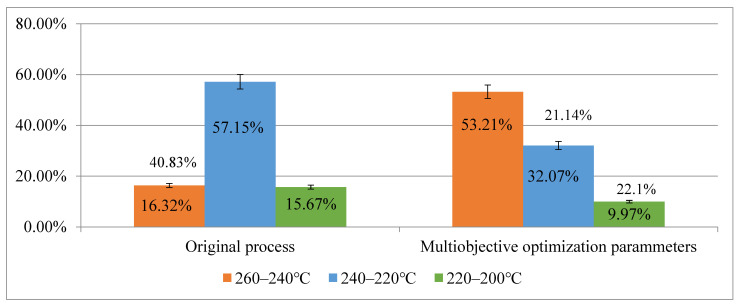
Comparison of Temperature Distribution Intervals.

Figure 10 presents a comparison of the temperature interval values. The figure shows that the main postfill average temperature range (220–240 °C) of the original process accounted for 57.15%, followed by the 260–240 °C range (16.32%), and the 200–220 °C range (15.67%). A significant difference was observed between the main temperature interval (220–240 °C) and the other two intervals. For the optimized process using the multiobjective optimization parameters, the main postfill average temperature range was 260–240 °C, which accounted for 53.21%, followed by 240–220 °C (32.07%). These two intervals collectively accounted for 85.28%. The temperature distribution variance of the optimized process was smaller than that of the other processes, and it reduced the likelihood of uneven cooling rates caused by large temperature fluctuations and minimized obvious injection flaws at the bonding sites due to warpage deformation.

### 3.4. Analysis and Comparison of Different Cooling Channel System Designs

**Figure 11:**
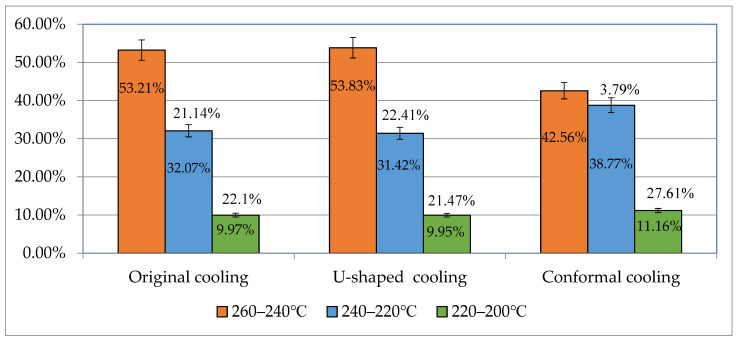
Comparison of Temperature Distribution Intervals.

**Figure 12:**
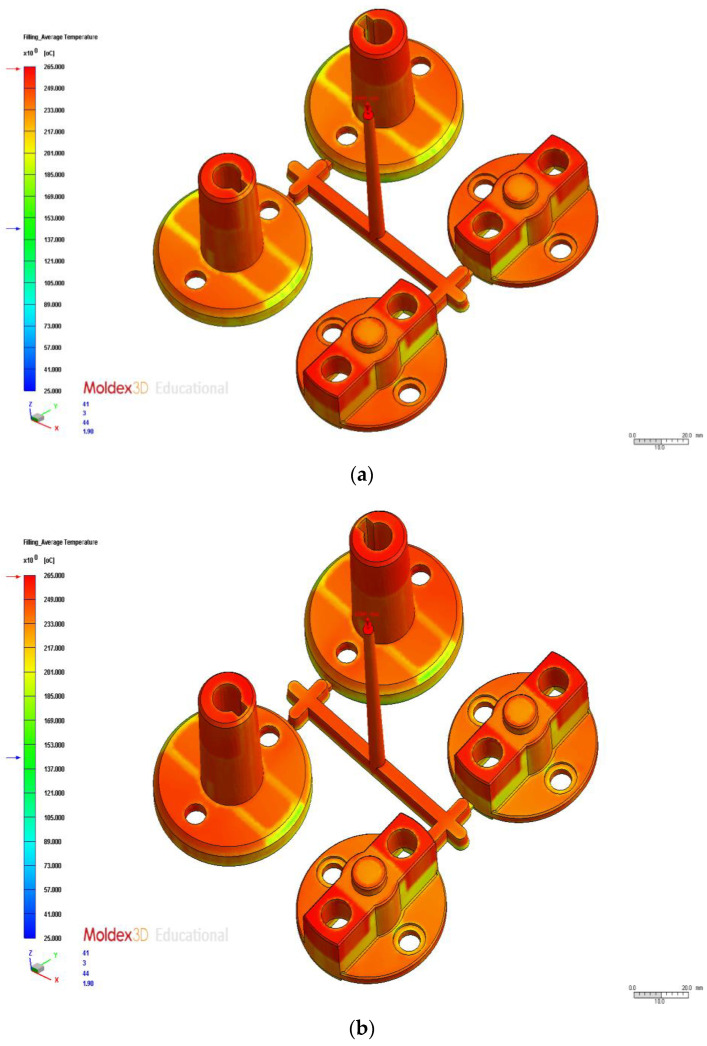

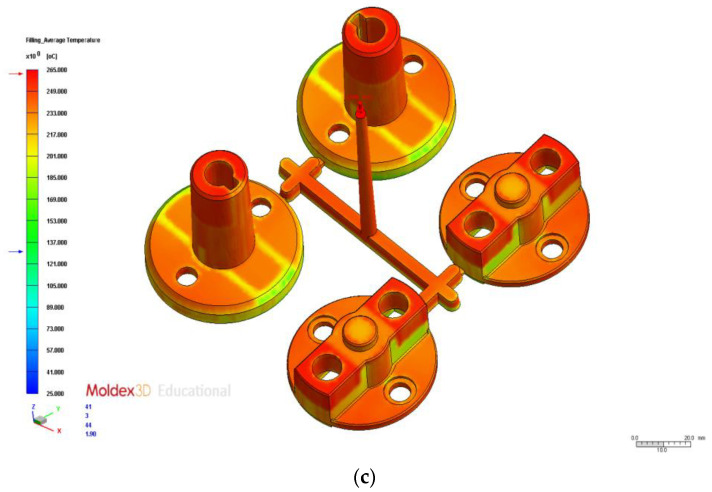
Comparison of Temperature Distributions. (**a**) Original cooling. (**b**) U-shaped cooling. (**c**) Conformal cooling.

**Figure 13:**
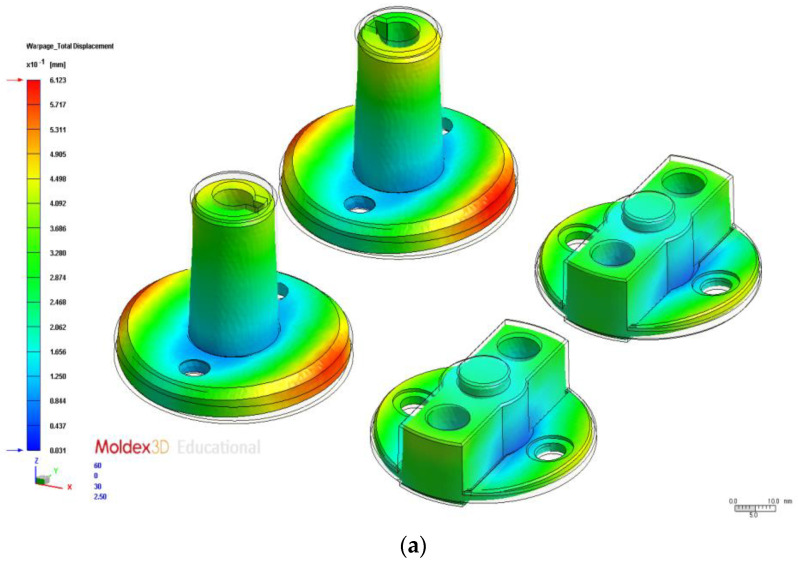

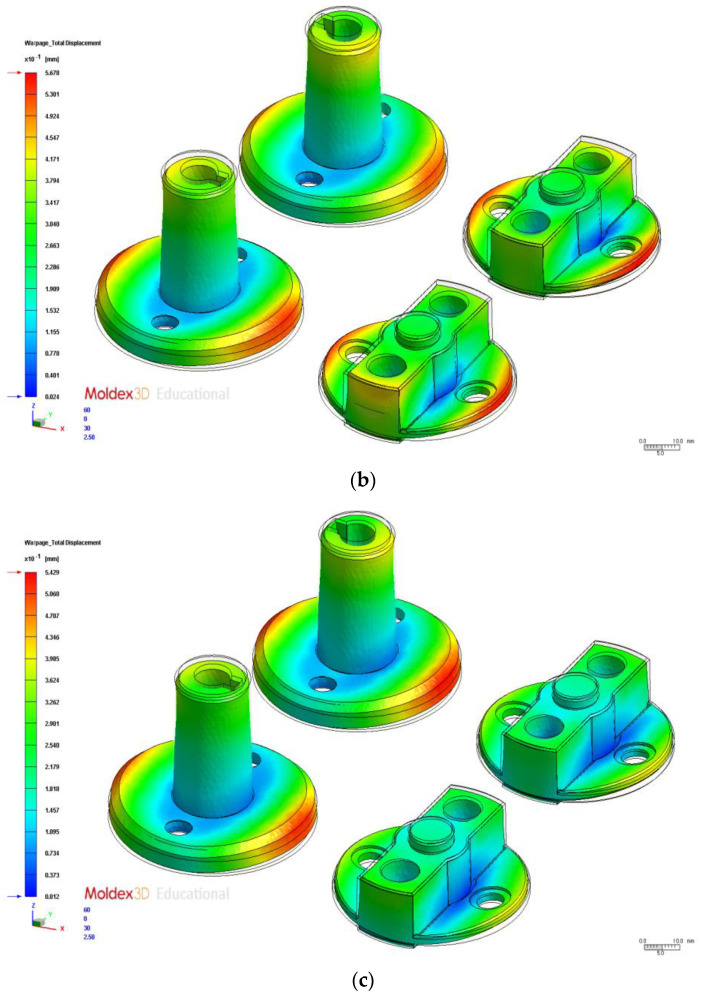
Comparison of Total Warpage. (**a**) Original cooling. (**b**) U-shaped cooling. (**c**) Conformal cooling.

In this section, we examine the effects of the process parameters obtained using multiobjective optimization (discussed in the previous section) on different cooling channel systems. We also compare the CAE analysis results of different cooling channel designs. The average temperature distribution differences with the original cooling, U-shaped cooling, and conformal cooling were 10.16 °C, 7.02 °C, and 5.78 °C, respectively. Figure 11 presents the postfill temperature distribution and their interval ranges for the three designs. Figure 12 displays the simulation results. The cooling channels in the conformal cooling design followed the shape of the auto lock parts. Therefore, the temperature differences were relatively low. Moreover, the temperature interval distribution of the conformal cooling design was the most favorable of the three designs. The postfill average temperature intervals of 240–260 °C and 220–240 °C accounted for 21.14%, 22.41%, and 3.79% in the original, U-shaped, and conformal cooling channel designs, respectively (Figure 11). These results confirmed that the conformal cooling channel design achieved more uniform temperatures and a smaller temperature interval distribution than the other designs. Therefore, this design reduces the likelihood of uneven cooling rates caused by fluctuating temperatures and warpage deformation caused by a nonuniform temperature distribution. We subsequently compared the effects of the three cooling channel designs on the warpage deformation. Figure 13 displays the three-axis displacement and overall displacement of the three cooling channel designs.

Table 18 lists the effects of the cooling channel designs on warpage deformation volume. The results indicated that the total warpage deformation of the conformal, U-shaped, and original cooling designs was 0.54 mm, 0.57 mm, and 0.61 mm, respectively, suggesting that the conformal cooling design coupled with parameters obtained using multiobjective optimization effectively reduced the warpage deformation volume.

## 4. Conclusions

In this study, we combined the Taguchi robust design method and gray relational analysis to assess the effects of various process parameters on the multiobjective optimization of the warpage and average temperature difference. We subsequently incorporated the parameters obtained using multiobjective optimization into different cooling channel designs and analyzed the differences. The findings were as follows:

1. The results of the Taguchi robust design tests combined with the optimization analyses indicated that the optimal parameter combination for warpage was A1B2C2D1E4, where A1 is injection time (0.6 s), B2 is material temperature (255 °C), C2 is mold temperature (75 °C), D1 is injection pressure (120 MPa), and E4 is holding pressure (145 MPa). The warpage was 0.61 mm; this was 0.29 mm less than that obtained using the original process parameters. The optimal parameter combination also enhanced the quality characteristics by 32.22%;
2. The results of the Taguchi robust design tests combined with the optimization analyses revealed that the optimal parameter combination for average temperature difference was A1B1C1D2E1, where A1 is injection time (0.6 s), B1 is material temperature (250 °C), C1 is mold temperature (65 °C), D2 is injection pressure (125 MPa), and E1 is holding pressure (130 MPa). The average temperature difference was 7.59 °C; this was 66.84 °C less than that obtained using the original process parameters. The optimal parameter combination also enhanced the quality characteristics by 47.40%;
3. Regarding the multiobjective optimization parameters obtained using the Taguchi gray relational analysis method, we adopted the smaller-the-better quality characteristics of the warpage and average temperature difference. The test results indicated that the multiobjective optimization parameter combination was A1B2C1D1E4, where A1 is injection time (0.6 s), B2 is material temperature (260 °C), C1 is mold temperature (65 °C), D1 is injection pressure (120 MPa), and E4 is holding pressure (140 MPa). The total warpage deformation volume was 0.62 mm, and the average temperature difference was 10.16 °C. Compared with the original parameter combination, the warpage deformation of the optimal parameter combination was 0.28 mm smaller, and the average temperature of the optimal parameter combination was 4.27 °C lower. To account for the multiobjective quality characteristics, we compared the results with the two single-objective optimal quality characteristics. We noted a 1.6% loss in warpage quality and a 5.2% loss in average temperature difference quality;
4. The results revealed that the warpage in the conformal cooling system was lower than the warpage in the other two systems. The conformal cooling system also improved the average temperature difference. The warpage was 0.54 mm, and the average temperature difference was 5.87 °C. These values are smaller than those of the original cooling system; specifically, the warpage and average temperature difference were reduced by 11.47% and 43.11%, respectively.

## Figures and Tables

**Table 1:** PA66 Material Characteristics.

Mechanical Properties	PA66
Density	1.14 (g/cc)
Poisson’s ratio	0.3
Modulus E	2 × 10^10^ (dyne/cm^2^)
CLTE	7.5 × 10^−5^ (1/K)
Fiber Weight Percentage	33 (%)
Percentage	275–305 °C
Melt Temperature	1.14 (g/cc)

**Table 2:** Original Process Parameters.

Factors	Level
A. Injection Time (s)	1
B. Material Temp. (°C)	245
C. Mold Temp.(°C)	65
D. Injection Press. (MPa)	120
E. Packing Press. (MPa)	130

**Table 3:** Comparison of Actual and Simulation Measurements of *Z*-axis Warpage Deformation.

Point	Actual Measurement (mm)	Simulation (mm)	Error (%)
1	0.50	0.54	1.08
2	−0.52	−0.62	1.19
3	0.39	0.44	1.13
4	0.59	0.48	0.81
5	−0.24	−0.26	1.08
6	0.55	0.42	0.76
7	0.48	0.50	1.04
8	−0.44	−0.61	1.34
9	0.23	0.49	2.13
10	0.46	0.47	1.02
11	−0.19	−0.26	1.37
12	0.43	0.42	0.98
AVG			1.16

**Table 4:** Control Factors and Levels.

Control Factors	Level			
	1	2	3	4
A. Injection Time (s)	0.6	0.8	1.0	1.2
B. Material Temp. (°C)	245	255	265	275
C. Mold Temp.(°C)	65	75	85	95
D. Injection Press. (MPa)	120	125	130	135
E. Packing Press. (MPa)	130	135	140	145

**Table 5:** Comparison of Different Water Cooling Systems.

Type	Number of Water Inlets	Number of Water Outlet	Reynolds Number	Cooling Liquid	Inlet Water Temperature
Original Cooling	2	2	6570	Oil	65 °C
U-shaped Cooling	6	6	6570	Oil	65 °C
Conformal Cooling	11	11	6570	Oil	65 °C

**Table 6:** S/N Ratios of Total Warpage.

No.	Warpage (mm)	S/N
1	0.69	3.27
2	0.65	3.80
3	0.64	3.82
4	0.65	3.72
5	0.63	4.00
6	0.64	3.83
7	0.67	3.48
8	0.73	2.79
9	0.71	2.92
10	0.71	3.01
11	0.63	3.95
12	0.69	3.25
13	0.74	2.70
14	0.62	4.08
15	0.70	3.09
16	0.71	3.02

**Table 7:** Total Warpage Deformation Response.

Factor	A	B	C	D	E
Level 1	3.65	3.22	3.52	3.51	3.04
Level 2	3.53	3.68	3.54	3.31	3.30
Level 3	3.28	3.59	3.40	3.46	3.40
Level 4	3.22	3.20	3.23	3.39	3.94
Effect	0.43	0.49	0.31	0.21	0.90
Rank	3	2	4	5	1
Optimal parameters	A1	B2	C2	D1	E4

**Table 8:** Confirmation of Total Warpage Deformation.

No.	Factor	Warpage (mm)
Original Process Parameters		0.90
Orthogonal Array Worst (No.13)	A4B1C4D3E2	0.74
Orthogonal Array Best (No.14)	A4B2C3D4E1	0.62
Optimization	A1B2C2D1E4	0.61

**Table 9:** Variance Analysis of Total Warpage Deformation.

Factor	DOF	Seq SS	MS	Contribution
A	3	0.49	0.16	14.92(%)
B	3	0.75	0.25	22.68(%)
C	3	0.24	0.08	7.31(%)
D			Pooled	
E	3	1.17	0.57	51.96(%)
Error	3	0.10	0.03	3.13(%)
Total	15	3.29		100(%)

**Table 10:** S/N Ratios of Average Temperature Difference.

No.	Average Temperature Difference (°C)	S/N
1	9.19	−19.27
2	11.28	−21.05
3	14.36	−23.14
4	16.74	−24.48
5	11.73	−21.39
6	14.12	−22.99
7	21.35	−26.59
8	12.33	−21.82
9	15.41	−23.75
10	16.66	−24.43
11	10.27	−20.23
12	11.42	−21.15
13	14.04	−22.95
14	14.00	−22.92
15	13.29	−22.47
16	11.52	−21.23

**Table 11:** Average Temperature Difference Response.

Factor	A	B	C	D	E
Level 1	−21.98	−21.84	−20.93	−22.48	−22.00
Level 2	−23.2	−22.85	−21.51	−21.51	−23.15
Level 3	−22.39	−23.11	−22.91	−22.55	−22.56
Level 4	−22.39	−23.17	−24.61	−23.42	−22.25
Effect	1.21	1.27	3.68	1.91	1.16
Rank	4	3	1	2	5
Optimal parameters	A1	B1	C1	D2	E1

**Table 12:** Average Temperature Difference Validation Test.

No.	Factor	Average Temperature Difference (°C)
Original process parameters		14.43
Orthogonal Array Worst (No.7)	A2B3C4D2E1	21.35
Orthogonal Array Best (No.1)	A1B1C1D1E1	9.19
Optimization	A1B1C1D2E1	7.59

**Table 13:** Variance Analysis of Average Temperature Difference.

Factor	DOF	Seq SS	MS	Contribution
A	3	3.10	1.03	6.23(%)
B	3	4.15	1.38	8.34(%)
C	3	32.21	10.74	64.70(%)
D	3	7.35	2.45	14.75(%)
E			Pooled	
Error	3	2.98	0.99	5.98(%)
Total	15	49.79		100(%)

**Table 14:** Variance Analysis of Average Temperature Difference.

No.	Warpage S/N Ratio	Average Temperature Difference S/N Ratio
1	1.00	0.70
2	0.86	0.87
3	0.73	0.88
4	0.67	0.84
5	0.83	0.96
6	0.74	0.89
7	0.59	0.77
8	0.80	0.61
9	0.70	0.63
10	0.68	0.65
11	0.91	0.93
12	0.85	0.71
13	0.74	0.59
14	0.75	1.00
15	0.77	0.67
16	0.84	0.66

**Table 15:** Gray Correlation Degree and Rank.

NO.	Gray Relation	Rank
1	0.85	5
2	0.86	4
3	0.81	7
4	0.76	9
5	0.90	2
6	0.82	6
7	0.68	13
8	0.71	12
9	0.67	14
10	0.66	16
11	0.93	1
12	0.78	8
13	0.67	15
14	0.87	3
15	0.72	11
16	0.75	10

**Table 16:** Multiobjective Response Form.

Factor	A	B	C	D	E
Level 1	0.82	0.77	0.86	0.80	0.73
Level 2	0.77	0.80	0.81	0.79	0.74
Level 3	0.76	0.77	0.76	0.78	0.77
Level 4	0.75	0.75	0.69	0.74	0.86
Effect	0.07	0.05	0.17	0.06	0.12
Rank	3	5	1	4	2
Optimal parameters	A1	B2	C1	D1	E4

**Table 17:** Multiobjective Optimization Comparison.

No.	Factor	Warpage (mm)	Average Temperature Difference (°C)
Original Process Parameters		0.90	14.43
Warpage Optimization	A1B2C2D1E4	0.61	
Average Temperature Difference Optimization	A1B1C1D2E1		7.59
Multi-Objective Optimization	A1B2C1D1E4	0.62	10.16

**Table 18:** Comparison of Warpage Deformation of Different Cooling Water Systems.

Warpage	Original Cooling	U-Shaped Cooling	Conformal Cooling
Total Warpage (mm)	0.61	0.57	0.54
X-Axis Warpage (mm)	−0.33~0.33	−0.29~0.29	−0.23~0.24
Y-Axis Warpage (mm)	−0.34~0.34	−0.41~0.42	−0.29~0.29
Z-Axis Warpage (mm)	−0.45~0.58	−0.43~0.54	−0.38~0.52

## Data Availability

The authors collected the data by themselves using the proposed method for this article.
